# Multi-omics integration reveals the role of PFOS in sarcopenia via ESRRG and RBP4: from computational prediction to experimental validation

**DOI:** 10.3389/fphar.2026.1926144

**Published:** 2026-10-08

**Authors:** Xingyao Yang, Xin Wang, Shuxing Xing, Zhangzhen Du

**Affiliations:** 1 Department of Orthopaedics, Chengdu Fifth People’s Hospital, Chengdu, China; 2 Department of Radiology, Medical Imaging Center, West China Second University Hospital, Sichuan University, Sichuan, China

**Keywords:** *in vitro* experiments, machine learning, PFOS, sarcopenia, single-nucleus RNA-seq

## Abstract

**Background:**

Perfluorooctane sulfonate (PFOS) has been shown to cause multi-organ dysfunction; however, the specific mechanisms underlying its association with sarcopenia remain unclear.

**Methods:**

Network toxicology analysis was performed to identify potential molecular targets and related signaling pathways involved in PFOS-induced sarcopenia. Molecular docking and dynamics simulations further screened stable PFOS–target interactions. Single-nucleus RNA-seq analysis was used to assess cell-type-specific PFOS-related signatures and ESRRG/RBP4-associated regulatory pathways in skeletal muscle. Finally, *in vitro* experiments were performed to evaluate the effects of PFOS on myogenic differentiation.

**Results:**

Network toxicology identified seven potential targets enriched in the PPAR signaling pathway. Molecular simulations indicated stable binding interactions between PFOS and ESRRG/RBP4. Single-nucleus RNA-seq analysis suggested that mast cells may represent a potential PFOS-responsive cell population and revealed cytoskeleton-related pathway alterations associated with ESRRG and RBP4. *In vitro* experiments further demonstrated that PFOS markedly inhibited myogenic differentiation, accompanied by downregulation of related protein expression.

**Conclusion:**

Our study demonstrates that exposure to PFOS is associated with an increased risk of sarcopenia and identifies ESRRG and RBP4 as key mediators of PFOS-associated myotoxicity.

## 1 Introduction

PFOS (perfluorooctane sulfonate) and PFOA (perfluorooctanoic acid) are representative members of the per- and polyfluoroalkyl substances (PFAS) family. These synthetic chemicals have been extensively applied in industrial and consumer products and are characterized by strong environmental persistence and resistance to degradation, thereby being classified as persistent organic pollutants (POPs) (Zuo et al., 2025; Suman and Kwak, 2025). Compared with PFOA, PFOS has had broader applications across multiple sectors, including textiles, leather processing, firefighting foams, electroplating, cleaning products, and electronics. As a result, humans may be exposed to PFOS through diverse routes, such as dermal contact and contaminated drinking water (Sevelsted et al., 2023). Accumulating evidence indicates that PFOS poses considerable risks to human health. Its toxicity involves complex and interconnected mechanisms of cellular injury, which may contribute to adverse effects in multiple organ systems, including the liver, kidneys, hematological system, and developmental and reproductive systems (Suman and Kwak, 2025).

Sarcopenia is a disorder characterized by progressive loss of skeletal muscle mass and function, leading to a series of unfavorable health outcomes (Cruz-Jentoft and Sayer, 2019). Although aging is a major contributor, sarcopenia is not exclusively age-related. Previous studies have shown that diabetes, Cushing’s syndrome, chronic kidney disease, and hepatic metabolic disorders may increase the risk of sarcopenia (Stangl et al., 2019). In terms of environmental toxicants, volatile organic compounds (VOCs) (Shi et al., 2025) and di(2-ethylhexyl) phthalate (DEHP) have been reported to be associated with sarcopenia progression (Hao et al., 2025). However, evidence regarding the potential relationship between PFOS exposure and sarcopenia remains limited.

Given the substantial health burden of sarcopenia and its public health implications (Jin et al., 2025; Yan et al., 2025), it is important to clarify whether persistent environmental pollutants, particularly PFOS, may contribute to the development of this condition. This study aimed to investigate whether PFOS affects skeletal muscle metabolism through endocrine disruption, metabolic dysregulation, or other potential mechanisms. To this end, we integrated multiple datasets and complementary approaches, including network toxicology, machine learning, molecular dynamics simulations, and cell-based experiments, to provide multi-level evidence and new insights into the prevention and treatment of sarcopenia (Figure 1).

**FIGURE 1:**
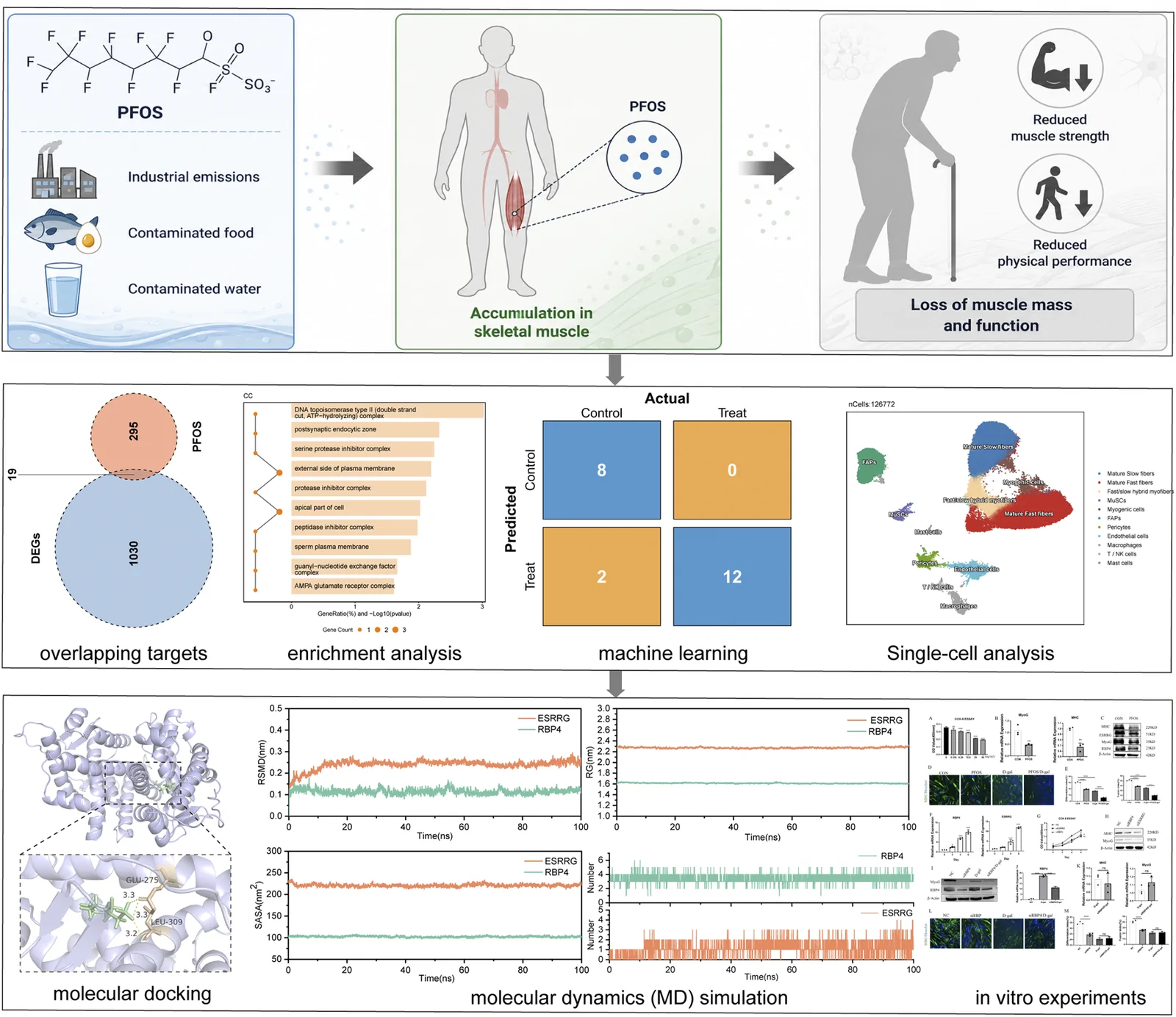
Research process.

## 2 Methods

### 2.1 Identification of potential targets for sarcopenia and PFOS

The GSE25941, GSE38718, and GSE9103 datasets (Supplementary Table S1) were retrieved from the GEO database (https://www.ncbi.nlm.nih.gov/geo/). Before differential expression analysis, the three datasets were integrated, and batch effects were corrected using the SVA package (version 3.5.0) (Leek et al., 2012; Chen B. et al., 2024). Principal component analysis (PCA) was then performed to evaluate the effectiveness of batch correction and assess data quality. After normalization, differential expression analysis between the sarcopenia and control groups was conducted using the Limma package. Differentially expressed genes (DEGs) were defined according to the thresholds of p < 0.05 and |log2FC| > 0.1. Volcano plots and heatmaps were generated using the ggplot2 R package to visualize the screening results.

The SMILES structure of PFOS was obtained from the PubChem database (https://pubchem.ncbi.nlm.nih.gov/). Potential PFOS-related targets were predicted using SwissTargetPrediction (http://swisstargetprediction.ch/), the SEA database (https://sea.bkslab.org/), and the SuperPred database (https://prediction.charite.de/subpages/target_prediction.php). The predicted targets were subsequently standardized using the STRING database (https://cn.string-db.org/).

### 2.2 Functional enrichment analysis of overlapping target genes

The Venn Diagram R package was used to identify overlapping genes between sarcopenia-related DEGs and PFOS-associated targets. These intersecting genes were then subjected to Gene Ontology (GO) and Kyoto Encyclopedia of Genes and Genomes (KEGG) enrichment analyses using the clusterProfiler package to investigate their potential biological functions and related signaling pathways. GO terms and KEGG pathways with p < 0.05 were considered statistically significant and retained for subsequent analyses.

### 2.3 Machine learning–based Identification and validation of key genes

To assess the diagnostic value of the identified target genes for sarcopenia, model performance was evaluated across 113 combinations generated from 12 machine learning algorithms. GSE25941, GSE38718, and GSE9103 were used as training datasets, whereas GSE1428 served as an independent external validation dataset and was not included in model training or feature selection. The 12 algorithms included Least Absolute Shrinkage and Selection Operator (LASSO), Ridge Regression, Stepwise Generalized Linear Model (StepGLM), eXtreme Gradient Boosting (XGBoost), Random Forest (RF), Elastic Net (Enet), Partial Least Squares Regression for Generalized Linear Models (PlsRglm), Generalized Boosted Regression Modeling (GBM), Naive Bayes, Linear Discriminant Analysis (LDA), Generalized Linear Model Boosting (GlmBoost), and Support Vector Machine (SVM).

To reduce the risk of overfitting, all models were trained using five-fold cross-validation within the training datasets. Feature selection and hyperparameter optimization were independently performed in each training fold through grid search to minimize information leakage. Model performance was assessed using the mean area under the receiver operating characteristic curve (AUC), and the final models were validated in GSE1428 (Chen B. et al., 2024; Qin et al., 2023), from which key genes were subsequently determined. Confusion matrices were used to evaluate the predictive performance of the models in both the training and external validation datasets. Finally, the Wilcoxon rank-sum test was applied to confirm the differential expression of machine learning-derived key genes between the normal and sarcopenia groups. The diagnostic performance of each key gene was further evaluated by calculating the AUC using the pROC package.

### 2.4 Molecular docking and molecular dynamics simulation

Molecular docking was performed to evaluate the binding affinity between hub genes and PFOS. Protein structures were obtained from the UniProt database (https://www.uniprot.org/) and the Protein Data Bank (https://www.rcsb.org/). The SDF file of PFOS was downloaded from PubChem and converted into a mol2 ligand file using Open Babel GUI. Ligand and receptor preparation was carried out using PyMOL, AutoDock Tools, and AutoDock Vina, and docking results were visualized with PyMOL (Liu et al., 2023).

Based on the docking results, molecular dynamics (MD) simulations of hub gene-PFOS complexes were conducted using GROMACS. Protein structures were repaired with SPDBV software, and small-molecule ligands were saved in mol2 format using PyMOL. Ligand and topology files were processed with Sobpob software. GROMACS was then used to generate protein and topology files, define the simulation box, add water molecules and ions, balance ionic strength, perform energy minimization, and conduct NVT equilibration. A 100-ns MD simulation was subsequently performed. After simulation, protein-ligand interaction stability was evaluated based on root mean square deviation (RMSD), radius of gyration (Rg), solvent-accessible surface area (SASA), and the number of hydrogen bonds (Hu et al., 2025).

### 2.5 SnRNA-seq analysis and hub gene virtual knockout

To characterize cellular heterogeneity in skeletal muscle during sarcopenia, single-nucleus RNA sequencing data from the GSE167186 cohort, comprising 6 young and 11 aged individuals, were reanalyzed using Seurat, with batch effects corrected by Harmony (Perez et al., 2022). After filtering low-quality nuclei and removing putative doublets, the top 2,000 highly variable genes were identified for subsequent analyses. Principal component analysis was performed for dimensionality reduction, and the cellular landscape was visualized using UMAP. Cell clustering was then conducted with the FindNeighbors and FindClusters functions at a resolution of 0.4.

Cell identities were assigned based on cluster-specific marker genes together with well-established canonical markers. The annotated cell populations included slow- and fast-twitch myofibers, satellite cells, myogenic cells, fibro-adipogenic progenitors, pericytes/vascular smooth muscle cells, endothelial cells, macrophages, T/NK cells, and mast cells.

Genes shared between PFOS-associated targets and sarcopenia-related differentially expressed genes were used to construct cell signature scores. Gene set activity across cell populations was quantified using multiple scoring approaches implemented in the irGSEA package (Fan et al., 2024), including AUCell, UCell, singscore, ssGSEA, and AddModuleScore. Cell subsets showing relatively high signature activity were further selected for cell-cell communication analysis using CellChat.

Furthermore, *in silico* knockout analysis of hub genes was performed with scTenifoldKnk to infer their potential regulatory effects at the single-cell level (Osorio et al., 2022). Genes predicted to be perturbed following virtual knockout were subsequently subjected to KEGG enrichment analysis, aiming to identify biological pathways potentially modulated by these hub genes.

### 2.6 Interaction between hub genes and toxicants

To further evaluate the relationships among the hub genes associated with sarcopenia, interaction analysis was performed using the Comparative Toxicogenomics Database (CTD). Through this analysis, chemical substances associated with the hub genes were identified and assessed (Huang et al., 2024).

### 2.7 *In vitro* experimental validation

C2C12 cells (Immocell Biotech, passages ≤5) were cultured at 60%–70% confluence and passaged 1:3. At ∼60% confluence, about 24 h post-seeding, differentiation was induced by DMEM (Gibco) containing 2% horse serum (Gibco) and 1% penicillin–streptomycin, with or without 40 g/L D-galactose or PFOS throughout the induction period. Medium was renewed every 2 days. qRT-PCR and Western blot were performed on day 4, and myotube staining on day 6 post-induction.

Total RNA was extracted from cells using TRIzol reagent. Specifically, cultured cells were lysed directly with TRIzol, while micromass and tissue samples were snap-frozen in liquid nitrogen and mechanically homogenized prior to extraction.For qRT-PCR analysis, 500 ng of total RNA was reverse-transcribed into cDNA using ReverTra Ace (Toyobo). qRT-PCR amplification was carried out in a 20 μL reaction system containing 1 μL of cDNA and SYBR FAST qRT-PCR Master Mix (Tsingke). Gene expression levels were quantified using the comparative Ct method (2^−ΔΔCT^) with β-actin as the endogenous control. All reactions were performed in triplicate. The primer sequences used are listed in Table 1.

**TABLE 1:** Primer sequences for the tested genes.

Gene	Forward primer	Reverse primer
RBP4	GTG​AGA​TGG​AGT​GGG​TGT​GG	CCC​AGA​GAA​ACG​AGC​CTT​GT
ESRRG	TGT​TAC​ATG​AGG​CCC​TGC​AG	CAG​CGT​CAT​CAG​CAT​CTT​GC
MHC	AGA​CGG​AGA​GGA​GCA​GGA​AGA​TTG	TTG​GTG​TTG​ATG​AGG​CTG​GTG​TTC
Myog	GTC​CCA​ACC​CAG​GAG​ATC​ATT​TGC	TCC​TCC​ACC​GTG​ATG​CTG​TCC

Proteins extracted with RIPA buffer were subjected to Western blot analysis. Briefly, samples were boiled, separated by SDS-PAGE, and transferred to a PVDF membrane. After blocking, membranes were incubated overnight at 4 °C with primary antibodies against RBP4 (proteintech 82934-1-RR,1:1000). ESRRG (proteintech 14017-1-AP, 1:1000). Myog (proteintech 26762-1-AP, 1:1000). MHC (Santa Cruz sc-376157, 1:500) followed by an HRP-conjugated secondary antibody. Signals were visualized using an ECL substrate. For gene knockdown, C2C12 cells were transfected with 50 nM siRNA using Lipofectamine 3000, and knockdown efficiency was confirmed by Western blot after 36 h before myogenic differentiation induction.

The siRNA sequences were as follows: siRBP4 Sense: GCA​UUG​AGA​UCA​UCA​ACA​UTT, Antisense: AUG​UUG​AUG​AUC​UCA​AUG​CTT. siESRRG Sense: GGA​UCU​ACC​UGA​GCU​CCA​ATT, Antisense: UUG​GAG​CUU​CAG​GUA​GAU​CCT​T.

Cells were seeded in a 96-well plate. After treatment, the CCK-8 reagent was added to each well according to the manufacturer’s instructions, followed by incubation at 37 °C for 1–4 h. Absorbance was measured at 450 nm using a microplate reader to determine cell viability.

On day 6 of differentiation, cells were processed for immunofluorescence staining. Briefly, cells were fixed with 4% paraformaldehyde for 30 min at room temperature, washed three times with PBS, and blocked with 5% bovine serum albumin (BSA) in PBS for 1 h. Subsequently, cells were incubated overnight at 4 °C with a primary antibody against myosin heavy chain (MHC; mouse monoclonal, Santa Cruz, sc-376157) diluted 1:100 in blocking solution. After washing with PBS, cells were incubated with an appropriate fluorophore-conjugated secondary for 2 h at room temperature in the dark. Nuclei were counterstained with Hoechst 33,342. Coverslips were mounted with antifade mounting medium and imaged using a fluorescence microscope.

Quantitative analysis was performed using ImageJ software. At least five random fields per condition were captured. The myogenic differentiation index was calculated as the percentage of MHC-positive nuclei relative to the total number of nuclei. The fusion index was defined as the percentage of nuclei contained within MHC-positive myotubes harboring three or more nuclei.

## 3 Results

### 3.1 Identification and enrichment analysis of relevant target genes

Using the “SVA” package, batch effects were effectively corrected (Figures 2A,B), enabling the successful integration of the three datasets—GSE25941, GSE38718, and GSE9103. A total of 1,149 DEGs associated with sarcopenia were identified and visualized through volcano and heat maps (Figures 2C,D). The Venn diagram revealed 19 overlapping genes shared between the sarcopenia-related DEGs and PFOS-associated target genes (Figure 2E). KEGG pathway analysis indicated that these 19 target genes were mainly enriched in the PPAR signaling pathway, proximal tubule bicarbonate reclamation, AMPK signaling pathway, and transcriptional misregulation in cancer (Figure 2F). In the GO analysis, the biological functions of the target genes were primarily associated with the hormone metabolic process and lipid catabolic process, mainly localized to the external side of the plasma membrane and the apical part of the cell, and involved in serine-type endopeptidase activity, serine-type peptidase activity, serine hydrolase activity, and carboxylic acid binding(Figure 2G).

**FIGURE 2:**
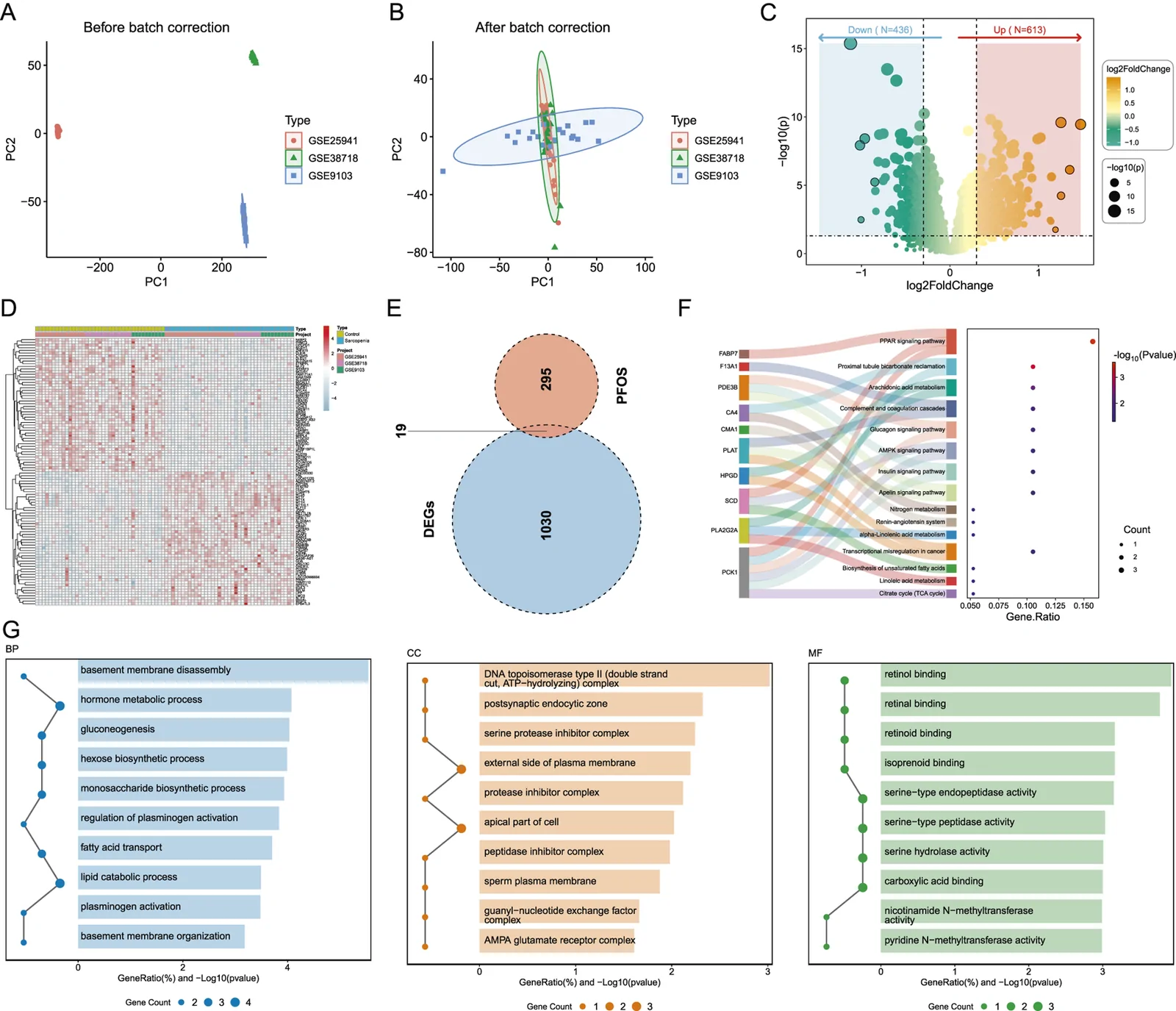
Identification of relevant target genes. **(A)** Before batch effect correction; **(B)** after batch effect correction; **(C)** volcano plot of DEGs in sarcopenia; **(D)** heatmap of DEGs in sarcopenia; **(E)** Venn diagram showing the intersection of related targets; **(F)** KEGG pathway enrichment analysis of target genes; **(G)** GO enrichment analysis of target genes, including cellular component (CC), molecular function (MF), and biological process (BP).

### 3.2 Identification of hub genes and construction of a disease prediction model

A total of 113 model combinations derived from 12 machine learning algorithms were used to identify the key genes (Figure 3A). The results indicated that the Random Forest (RF) model was the most reliable. The RF model identified seven key genes: CA4, DUSP3, ESRRG, HPGD, PLAT, RBP4, and SCD (Figure 3A). The area under the curve (AUC) of the RF model was 0.999 in the training dataset and 0.975 in the external validation dataset, with an average AUC of 0.988 (Figure 3B). The ROC analysis demonstrated that the key genes selected by the RF model possessed high discriminative value in distinguishing sarcopenia samples from normal samples, as reflected by the excellent AUC values in both the training and validation cohorts (Figure 3B). The confusion matrix further confirmed that the model exhibited strong accuracy and sensitivity in both datasets (Figure 3C). Moreover, the expression levels of these seven key genes showed significant differences between sarcopenia and normal samples (Figure 3D). In addition, all seven hub genes displayed favorable predictive performance for disease occurrence, with AUC values greater than 0.7.

**FIGURE 3:**
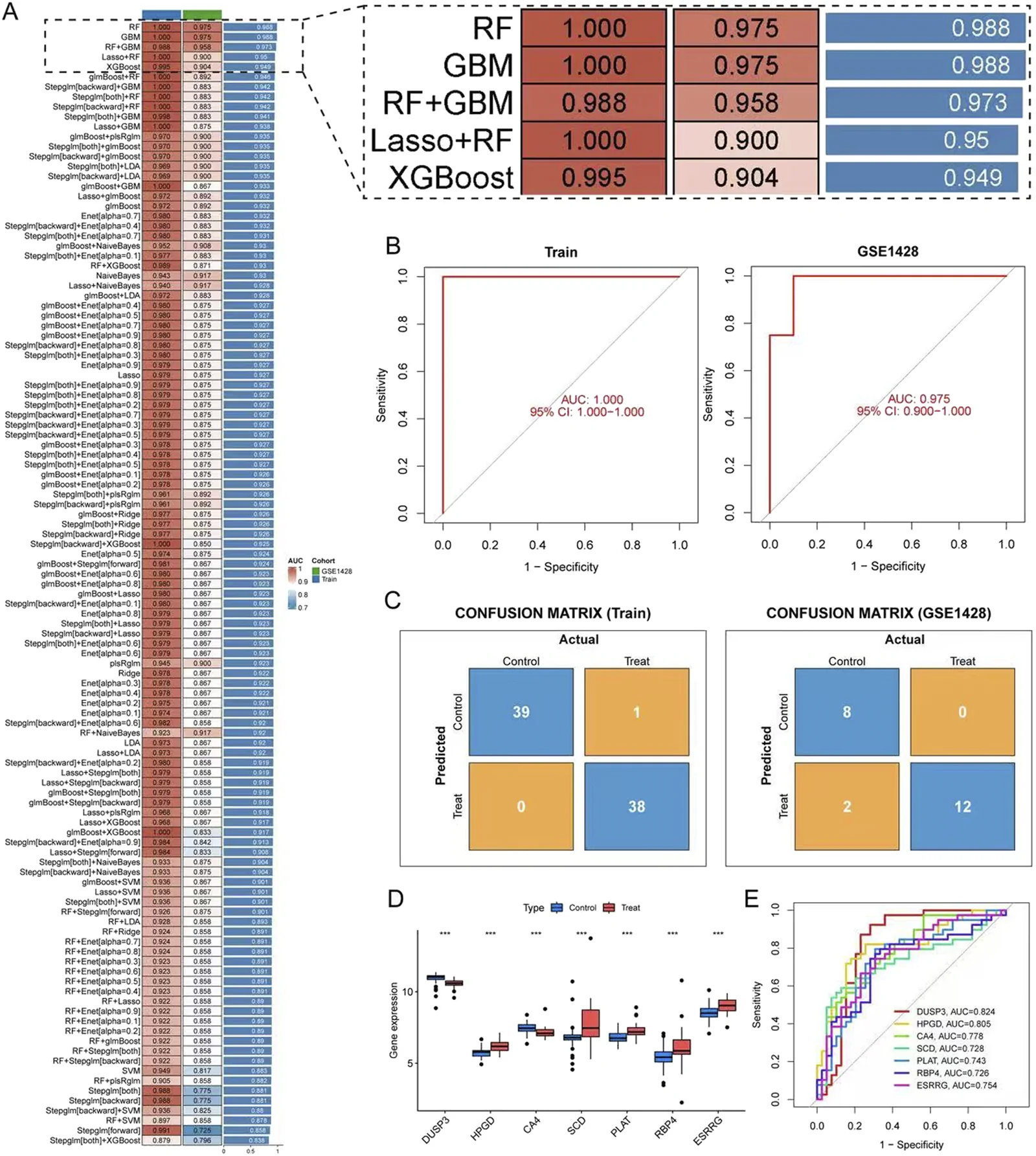
Identification of hub genes. **(A)** Identification of key genes based on 113 combinations of machine learning algorithms; **(B)** ROC curves of the optimal Random Forest (RF) model in the training and external validation datasets; **(C)** confusion matrices of the RF model in the training and validation cohorts; **(D)** differential expression of key genes between sarcopenia and normal samples; **(E)** predictive performance of key genes for disease occurrence.

### 3.3 Molecular docking and molecular dynamics simulation

Molecular docking was conducted to elucidate the binding interactions between PFOS and the proteins encoded by the identified hub genes. The binding energies of PFOS with the seven target proteins are presented in Figure 4A. A binding energy lower than −7 kcal/mol generally indicates strong ligand–protein affinity. In this study, ESRRG (−9.10 kcal/mol) and RBP4 (−10.50 kcal/mol) exhibited the strongest affinities for PFOS and were selected for subsequent analyses. Both proteins displayed binding energies below −9.0 kcal/mol, suggesting highly stable interactions. PFOS was predicted to interact with these proteins through multiple noncovalent forces, including hydrophobic interactions, van der Waals forces, hydrogen bonds, and halogen bonds.

**FIGURE 4:**
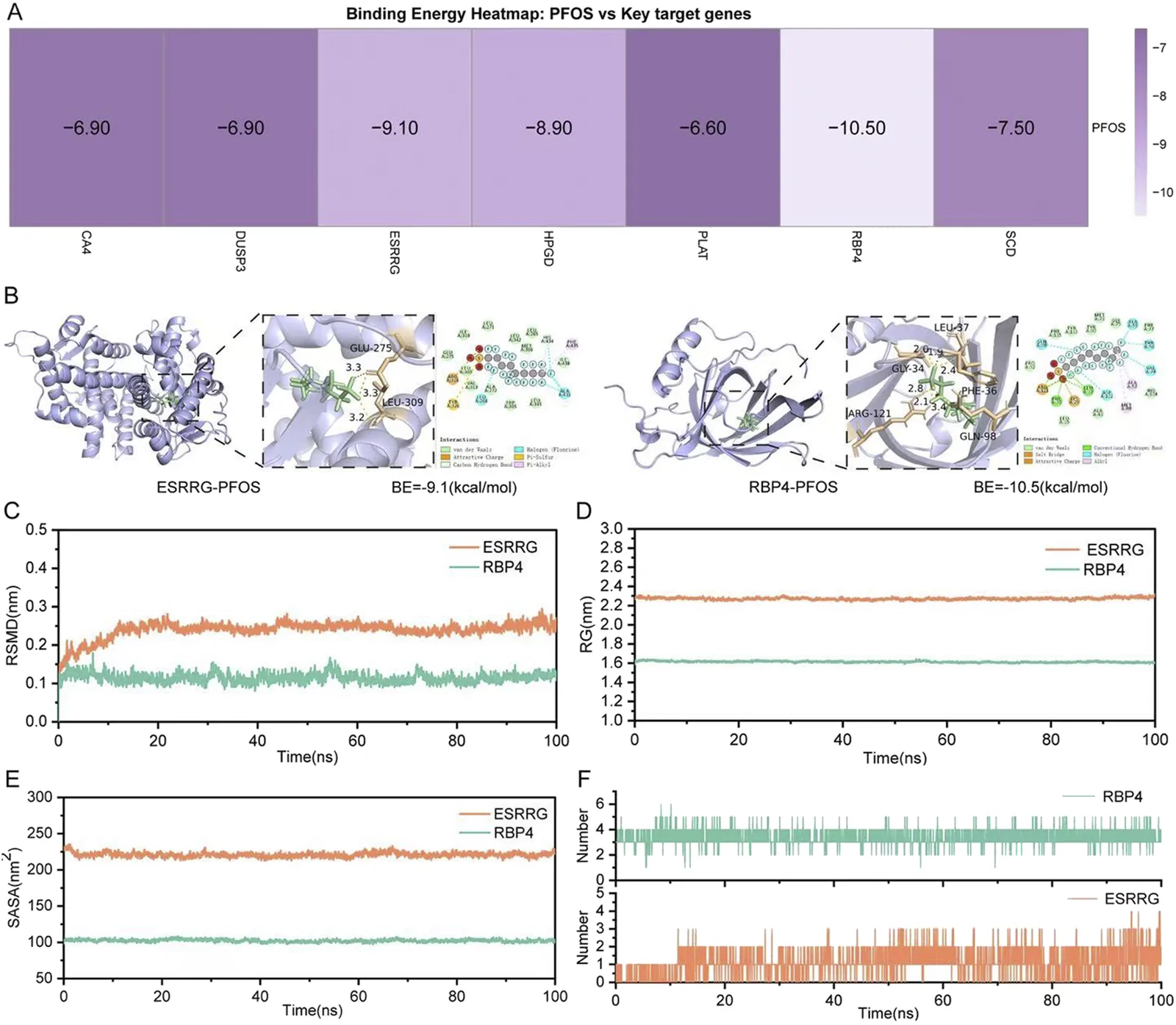
Molecular docking analysis. **(A)** Binding energies between PFOS and hub gene–encoded proteins; **(B)** Docking conformations of PFOS with ESRRG and RBP4; **(C)** Root mean square deviation (RMSD); **(D)** radius of gyration (Rg); **(E)** solvent-accessible surface area (SASA); **(F)** number of hydrogen bonds.

To further assess the dynamic stability of these complexes, molecular dynamics (MD) simulations were performed for the ESRRG–PFOS and RBP4–PFOS systems. The root mean square deviation (RMSD) was used to evaluate conformational stability, where RMSD values within 1 nm indicate that a complex remains structurally stable under physiological conditions (Figure 4C). Throughout the simulation, both the radius of gyration (Rg) and solvent-accessible surface area (SASA) exhibited minimal fluctuations, implying only slight conformational adjustments without notable structural disruption (Figures 4D,E). Hydrogen bond analysis revealed that the ESRRG-PFOS complex maintained 0–4 hydrogen bonds, while the RBP4-PFOS complex formed 1–6, with those in the latter being more frequent and stable (Figure 4F). Collectively, these MD results confirm that both ESRRG–PFOS and RBP4-PFOS complexes possess stable and persistent binding interactions.

### 3.4 Cell-type specific effects of PFOS-associated signatures in skeletal muscle

UMAP projection of the integrated snRNA-seq dataset resolved 11 transcriptionally distinct cell clusters with clear separation (Figure 5A). The feature expression patterns of canonical marker genes showed cluster-specific enrichment, supporting reliable annotation of the major skeletal muscle cell types (Figure 5B). Cell composition analysis revealed a relative reduction in mature slow-twitch fibers and myogenic cells in aged samples, whereas skeletal muscle satellite cells (MuSCs) and endothelial cells were proportionally increased compared with the young group (Figure 5C).

**FIGURE 5:**
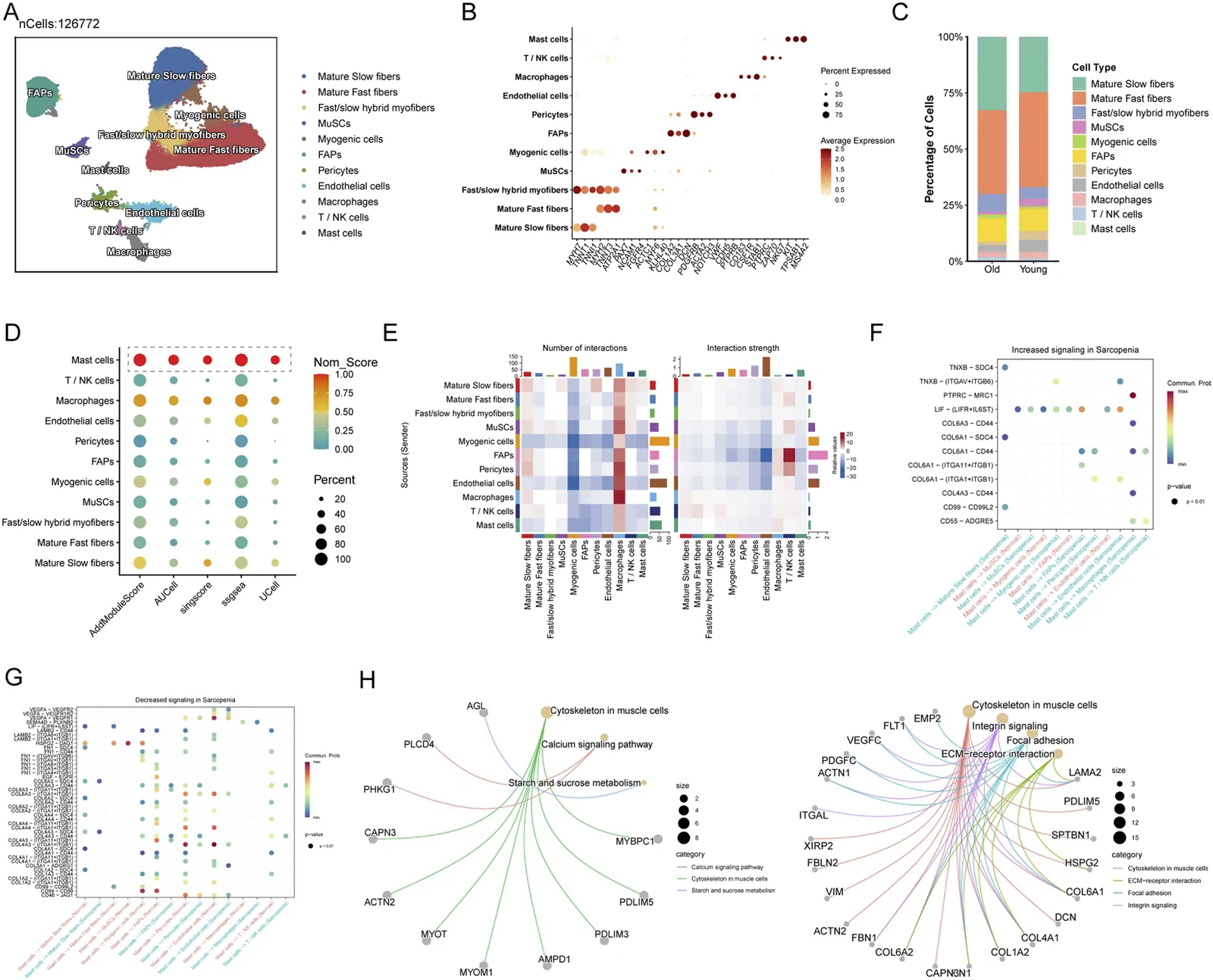
Single-nucleus RNA-seq analysis, cell–cell communication, and virtual knockout analysis in skeletal muscle. **(A)** UMAP of 11 cell types. **(B)** Canonical marker gene expression across cell types. **(C)** Cell type composition in young and aged groups. **(D)** PFOS-related gene signature scores across cell types. **(E)** CellChat heatmap showing overall differences in cell–cell communication networks between groups. **(F)** Increased mast cell–centered signaling in sarcopenia. **(G)** Decreased mast cell–centered signaling in sarcopenia. **(H)** KEGG enrichment of genes affected by ESRRG and RBP4 virtual knockout.

PFOS-related gene set activity, evaluated using five independent computational approaches, consistently indicated that mast cells exhibited the highest signature scores among all annotated cell types (Figure 5D). Subsequent cell-cell communication analysis demonstrated that mast cells acted as signaling senders and macrophages as receivers, with both the interaction number and interaction strength increased in aged muscle (Figure 5E). Ligand-receptor analysis further revealed enhanced PTPRC-MRC1 signaling from mast cells to macrophages in aged samples (Figure 5F). In contrast, HSPG2-DAG1-mediated signaling between mast cells and MuSCs was attenuated. Similarly, COL4A3-(ITGA1+ITGB1) interactions involving mast cells with both pericytes and endothelial cells were weakened in the aged group (Figure 5G).

In silico knockout analysis identified 18 and 180 significantly altered downstream genes following ESRRG and RBP4 perturbation, respectively (P < 0.05). KEGG enrichment analysis of these perturbed genes highlighted “cytoskeleton in muscle cells” as the most significantly enriched pathway (Figure 5H).

### 3.5 Interaction analysis between core target genes and related toxicants

ESRRG and RBP4 were identified as core target genes linking PFOS exposure to sarcopenia. To explore their toxicological relevance, interactions were retrieved from the Comparative Toxicogenomics Database (CTD). Of the 127 toxicants associated with ESRRG and 176 with RBP4, 47—including PFOS—were shared by both genes, indicating potential environmental factors that may contribute to their dysregulation (Figure 6).

**FIGURE 6:**
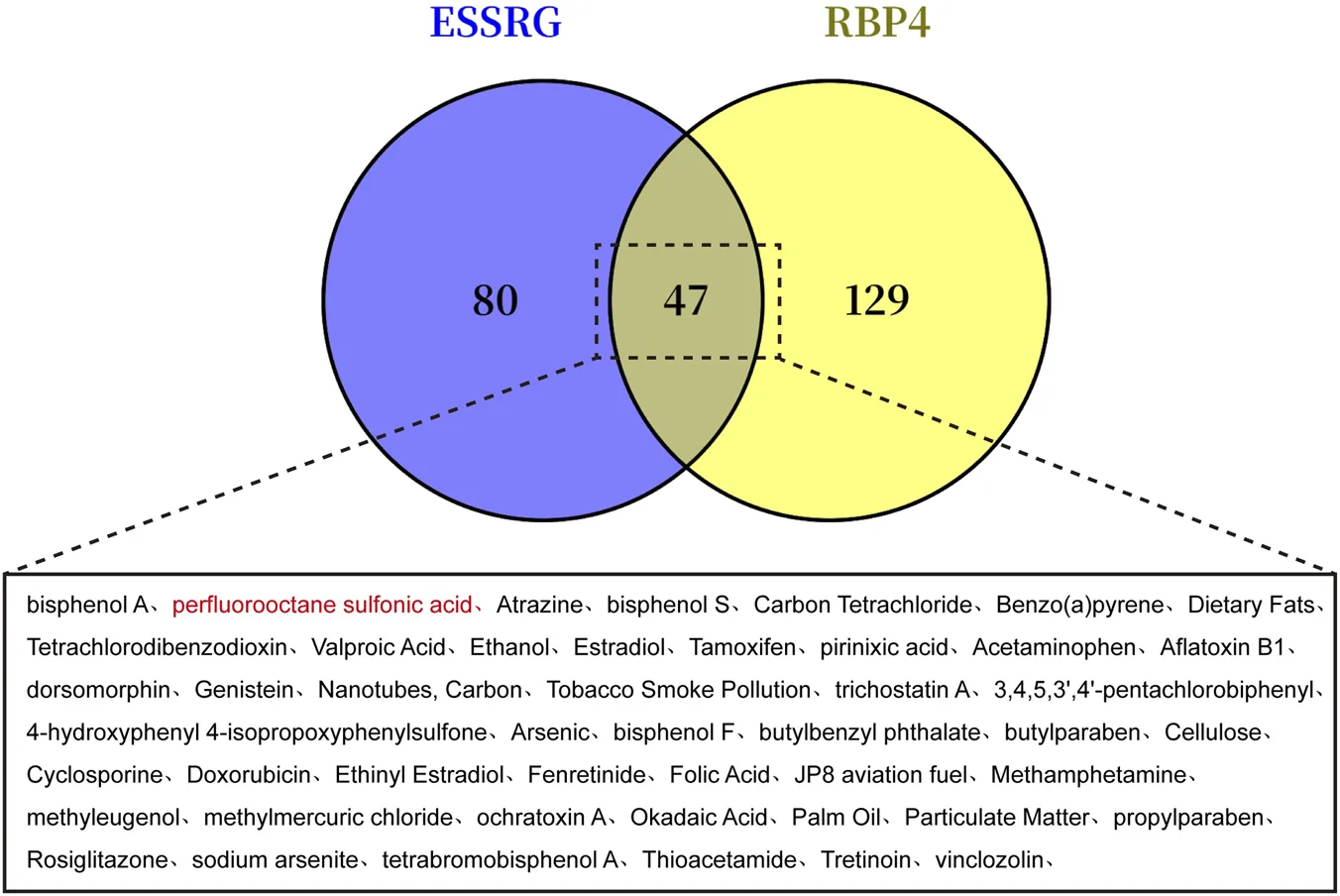
Venn diagram showing the overlapping toxicants interacting with ESRRG and RBP4.

### 3.6 *In vitro* validation: PFOS impairs myogenic differentiation via downregulation of RBP4 and ESRRG

To further investigate the role of PFOS in sarcopenia, we utilized the C2C12 cell line as a model system. Initial CCK-8 assays revealed that PFOS significantly inhibited myoblast proliferation, with a pronounced effect observed at a concentration of 25 ng/ml (Figure 7A). We subsequently examined its impact on C2C12 myogenic differentiation. Experimental results demonstrated that 25 ng/ml PFOS effectively suppressed the expression of both myogenin (MyoG) and myosin heavy chain (MHC) during differentiation (Figures 7B,C).

**FIGURE 7:**
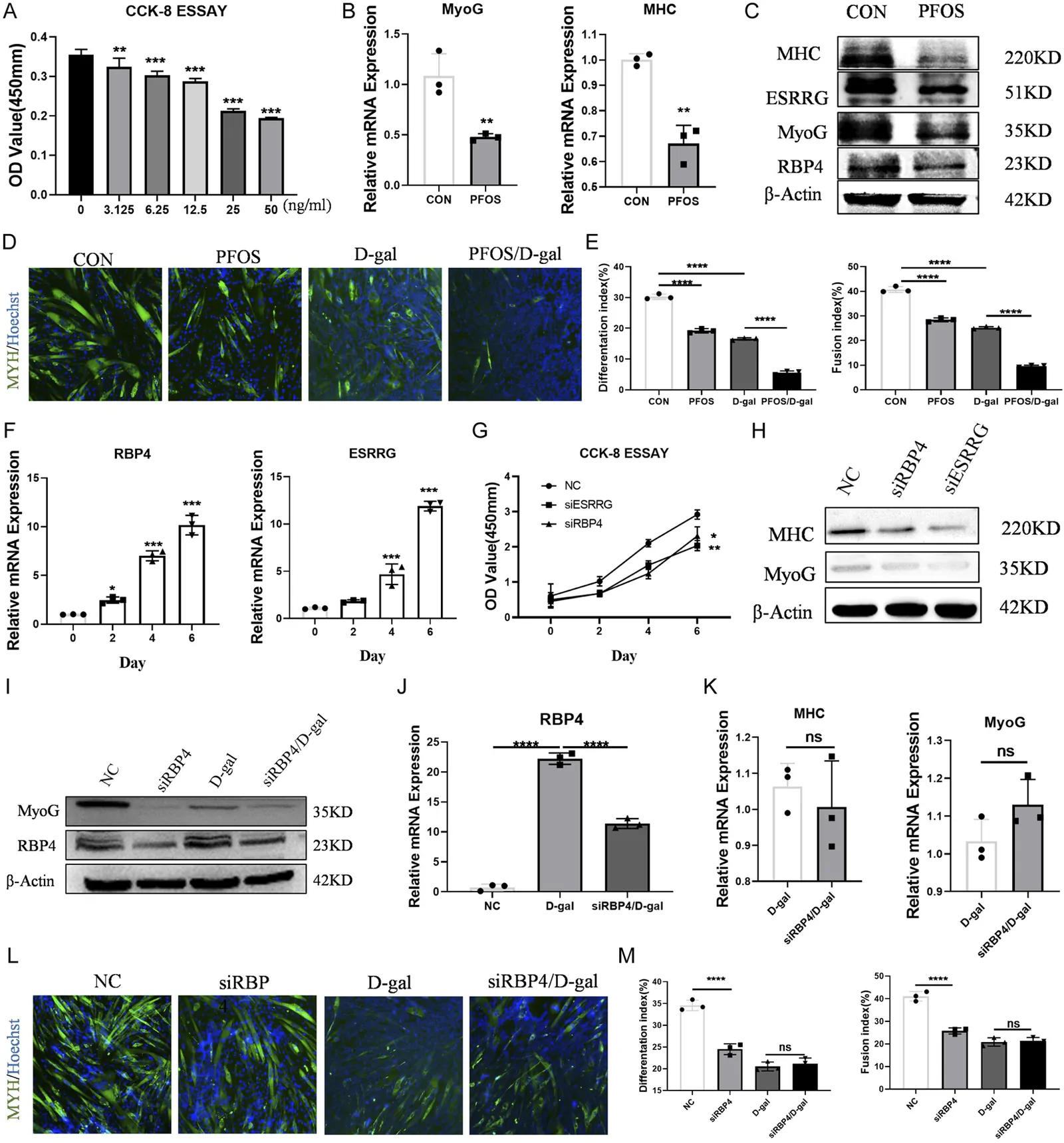
PFOS Impairs Myogenic Differentiation via Downregulation of RBP4 and ESRRG. **(A)** CCK-8 assay demonstrating that PFOS inhibits C2C12 myoblast proliferation after 8-h exposure (n = 5). **(B,C)** PFOS treatment suppresses the expression of myogenic markers (Myogenin and myosin heavy chain) and candidate genes (RBP4 and ESRRG) during C2C12 differentiation (CON: normal differentiation; PFOS: differentiation with PFOS treatment). **(D,E)** Representative immunofluorescence images (D, captured using a ×20 objective) and quantitative analysis **(E)** of myotube formation and fusion index under basal and D-gal-induced senescence conditions, with or without PFOS. PFOS consistently impaired differentiation in both settings (n = 3). **(F)** Expression dynamics of RBP4 and ESRRG during normal myogenic differentiation (n = 3). **(G)** Silencing of RBP4 or ESRRG inhibits C2C12 cell proliferation (n = 5). **(H)** Silencing of RBP4 or ESRRG impairs myogenic differentiation, as indicated by reduced myogenic protein expression (NC: differentiation after transfection with scrambled RNA; siRBP4/siESRRG: differentiation after transfection with corresponding siRNA). **(I)** Western blot analysis of MyoG expression with or without RBP4 knockdown under D-gal-induced senescence. **(J)** qRT-PCR analysis of RBP4 expression and its knockdown efficiency under D-gal treatment (n = 3). **(K)** qRT-PCR analysis of MHC and MyoG gene expression following RBP4 knockdown under D-gal-induced senescence (n = 3). **(L,M)** Representative immunofluorescence images (L, captured using a ×20 objective) and quantitative analysis **(M)** of myotube formation efficiency and fusion index in C2C12 cells under basal or D-gal-induced senescence conditions, with or without RBP4 knockdown (n = 3). *All quantitative data are presented as mean ± SEM from at least three independent experiments. Statistical significance was determined using Student’s t-test (for two groups) or one-way ANOVA (for three or more); *p < 0.05, **p < 0.01, ***p < 0.001, ****p < 0.0001.

Given that sarcopenia predominantly occurs in the context of organismal aging, we further validated the effect of PFOS in a D-galactose (D-gal)-induced cellular senescence model. Immunofluorescence results indicated that PFOS inhibited C2C12 myogenic differentiation under both basal conditions and D-gal-induced senescence, primarily manifested as significantly reduced myotube formation efficiency and fusion index (Figures 7D,E). These findings are consistent with our earlier experimental observations.

Based on prior analyses, we hypothesized that PFOS might exert its effects via the RBP4 and ESRRG genes. To test this hypothesis, qRT-PCR was performed. The results showed that expression levels of both RBP4 and ESRRG were elevated during normal myogenesis (Figure 7F). However, in the presence of PFOS, the expression of these two genes was significantly suppressed (Figure 7C). To further validate the functional role of RBP4 and ESRRG in myogenesis, we knocked down each gene individually in C2C12 cells using siRNA. The results indicated that depletion of either RBP4 or ESRRG impaired both cell proliferation and myogenic differentiation capacity (Figures 7G,H).

It is noteworthy that previous studies on the correlation between RBP4 and sarcopenia have reported elevated RBP4 levels in patients, and inhibition of RBP4 appears to be beneficial in mitigating muscle aging (Zhang K. Z. et al., 2024; Chang et al., 2023; Freitas et al., 2024).

This observation seems contradictory to our conclusions. To further explore this discrepancy, we investigated the role of RBP4 during C2C12 myogenesis in the senescence model. The results demonstrated that under normal differentiation conditions, knockdown of RBP4 significantly inhibited myogenesis, as evidenced by decreased expression of myogenic markers and reduced differentiation efficiency (Figures 7H–J). However, under D-gal-induced senescence, the inhibitory effect of RBP4 knockdown appeared attenuated. This was primarily manifested by an insignificant decrease in both the gene and protein expression levels of MyoG (Figures 7I,K), as well as no significant differences observed in MHC gene expression (Figure 7K), myotube formation efficiency, or fusion index (Figures 7L,M). Concurrently, we observed that RBP4 protein levels were markedly increased during differentiation in the presence of D-gal (Figures 7I,J), which aligns with previous reports. This suggests that the role of RBP4 in C2C12 cells may be context-dependent, and the underlying mechanisms require further elucidation. Nevertheless, our results clearly indicate that under normal physiological conditions, PFOS inhibits C2C12 myogenic differentiation, at least in part, through the downregulation of RBP4.

## 4 Discussion

With the acceleration of global population aging, sarcopenia has become an increasingly important public health issue, contributing to higher risks of falls, fractures, disability, hospitalization, and mortality (Almohaisen et al., 2022; Zhang et al., 2018). Sarcopenia has become a major public health issue affecting the health and quality of life of older adults (Chen L. K. et al., 2022). Epidemiological studies have shown that the prevalence of sarcopenia among community-dwelling older adults ranges from approximately 10%–30% and increases significantly with advancing age (Kirk et al., 2020).

In addition to classical risk factors such as physical inactivity, malnutrition, smoking, abnormal sleep duration, and diabetes (Freitas et al., 2024), emerging evidence suggests that environmental endocrine-disrupting chemicals may also participate in sarcopenia development (Yuan and Larsson, 2023). Previous studies have demonstrated that PFOS can disrupt the intestinal barrier (Zhang B. et al., 2024), impair thyroid function (Coperchini et al., 2017), reduce fertility (Chen Z. et al., 2024), and damage renal function (Zuo et al., 2025), Moreover, PFOS has been detected in the blood of women with pregnancy-related complications (Hofmann et al., 2023). However, whether PFOS exposure is associated with sarcopenia and the underlying mechanisms remain insufficiently understood.

In this study, we integrated network toxicology, transcriptomic datasets, machine learning, molecular docking, molecular dynamics simulations, snRNA-seq analysis, and *in vitro* experiments to explore the potential relationship between PFOS exposure and sarcopenia. Through these multi-level analyses, ESRRG and RBP4 were identified as key hub genes potentially linking PFOS exposure to sarcopenia-related muscle dysfunction. Subsequent cellular experiments further showed that PFOS exposure impaired C2C12 myogenic differentiation, accompanied by altered expression of ESRRG and RBP4. These findings support a potential mechanistic connection between PFOS exposure and impaired skeletal muscle formation.

Functional enrichment analysis indicated that the identified targets were mainly involved in the PPAR signaling pathway, proximal tubular bicarbonate reclamation, AMPK signaling pathway, and transcriptional misregulation in cancer. The PPAR signaling pathway is closely related to lipid metabolism, mitochondrial function, and metabolic homeostasis in skeletal muscle (Manickam et al., 2020). Proximal tubular bicarbonate reclamation is essential for renal acid-base regulation, and its impairment may indirectly contribute to muscle wasting through systemic metabolic disturbances (Visser et al., 2023). The AMPK signaling pathway is a central regulator of energy metabolism and inflammation, and its balance is critical for maintaining skeletal muscle mass (Hardman et al., 2014). Although transcriptional misregulation in cancer is primarily associated with tumor-related transcriptional abnormalities, cancer-related inflammatory mediators such as IL-6 and TNF-α may promote muscle catabolism and cachexia (Rao et al., 2022). These enriched pathways collectively suggest that PFOS may influence sarcopenia through metabolic, inflammatory, and systemic regulatory mechanisms.

SnRNA-seq analysis provided further cell-type-specific evidence for the potential role of PFOS-related signatures in skeletal muscle aging. Eleven skeletal muscle cell populations were identified, reflecting the cellular heterogeneity of the muscle microenvironment. Notably, PFOS-related gene signatures were most prominently enriched in mast cells rather than being evenly distributed across all cell types. Cell-cell communication analysis further suggested that aging was accompanied by remodeling of mast cell-centered intercellular signaling networks involving macrophages, MuSCs, myogenic cells, pericytes, and endothelial cells. In particular, immune-related signaling such as PTPRC-MRC1 was enhanced, whereas extracellular matrix- and adhesion-related interactions, including HSPG2-DAG1 and collagen-integrin signaling, were attenuated. These results suggest a shift from regenerative-supportive communication toward a more inflammatory microenvironment in aged muscle.

In silico perturbation analysis further showed that ESRRG and RBP4 may regulate genes involved in muscle cytoskeleton organization, while RBP4 was also associated with focal adhesion and ECM-receptor interaction pathways. These findings indicate that PFOS-related hub genes may affect skeletal muscle homeostasis not only through immune microenvironment remodeling but also through disruption of structural and regenerative signaling. Previous studies have shown that mast cells can be activated during skeletal muscle injury, regeneration, and aging and may participate in cancer-associated cachexia (Widner et al., 2021). which is consistent with our snRNA-seq findings. In addition, reverse toxicant prediction again identified PFOS as a potential compound associated with ESRRG and RBP4, further supporting the robustness of these hub genes.

ESRRG (also known as ERRγ), a member of the estrogen-related receptor family, functions as a key regulator of skeletal muscle metabolism. This nuclear receptor orchestrates a multifaceted optimization of skeletal muscle energy output, oxidative stress response, and contractile function by driving gene networks involved in mitochondrial biogenesis, oxidative metabolism, slow-twitch fiber specification, angiogenesis, and calcium homeostasis (Rangwala et al., 2010). Pathological relevance is established in sarcopenia research, where diminished ESRRG expression in aged muscle coincides with mitochondrial dysfunction, suggesting its role as a pivotal contributor to the decline in muscle strength (Shu et al., 2023). The role of ESRRG extends to systemic regulation: by modulating local metabolic status and vascular function, it provides nutritional and hemodynamic support to muscle tissue. This supportive milieu also serves as a foundation for effective satellite cell-mediated regeneration and the maintenance of muscle mass (Wang et al., 2025). However, a translational gap remains between mechanistic understanding and clinical validation. Although the ERR family is expressed in multiple metabolic organs (e.g., heart, liver), robust large-scale interventional studies are still lacking to demonstrate that specific activation of ESRRG can safely and significantly reverse age-related muscle loss and functional decline (Chen Q. et al., 2022).

Retinol-binding protein 4 (RBP4), a pro-inflammatory adipokine, has been increasingly recognized as a contributor to sarcopenia pathophysiology. Studies have shown that RBP4 knockout attenuates muscle wasting in denervation-induced atrophy models, and clinical cohorts have consistently linked elevated serum RBP4 levels to sarcopenia severity and future muscle loss in older adults (Zhang K. Z. et al., 2024; Chang et al., 2023; Freitas et al., 2024). These deleterious effects are thought to be mediated through RBP4-induced insulin resistance, NLRP3 inflammasome activation, and mitochondrial dysfunction (Graham et al., 2006; Wang et al., 2023; Wang et al., 2015).

However, our results suggest that the role of RBP4 in muscle biology may be context-dependent. During normal C2C12 myogenic differentiation, RBP4 expression was physiologically increased, and RBP4 knockdown impaired myotube formation and myogenic marker expression, indicating that endogenous muscle-derived RBP4 may support myogenesis under healthy regenerative conditions. In contrast, under D-galactose-induced senescence, RBP4 expression was pathologically elevated, but its knockdown failed to restore myogenic differentiation, suggesting that RBP4 may be overridden by stronger inhibitory networks in the senescent microenvironment.

This apparent discrepancy may be explained by the different biological sources and contexts of RBP4. Circulating RBP4, mainly derived from adipose tissue and the liver, may act as a systemic mediator of inflammation and metabolic dysfunction, whereas muscle-derived RBP4 may have a local supportive role during myogenic differentiation when maintained at an appropriate level. Therefore, RBP4 may function as a context-dependent regulator in skeletal muscle: beneficial for regeneration under young or healthy conditions, but detrimental or secondary to other pathological processes in aged or diseased states. This distinction may be important when considering RBP4 as a potential therapeutic target in sarcopenia.

Several limitations should be acknowledged. First, although C2C12 myoblasts are widely used for studying myogenesis, they cannot fully recapitulate authentic aging or sarcopenic phenotypes. The D-galactose-induced senescence model used in this study mainly reflects oxidative stress-related premature aging and does not represent the full complexity of organismal aging. Second, the PFOS concentration of 25 ng/mL was selected based on dose-response screening to induce reproducible phenotypic changes. Although this concentration is within or close to the upper exposure range reported in certain populations, it is higher than the average exposure level in the general U.S. population. Therefore, the present study should be interpreted as a mechanistic investigation rather than a quantitative risk assessment. Third, PFOS pharmacokinetics and potential sex-, age-, and ethnicity-related differences were not assessed. *In vivo* experiments and validation in human primary myoblasts or aged animal models are needed to confirm the causal relationship and translational relevance. Finally, the upstream mechanisms by which PFOS regulates ESRRG and RBP4, as well as additional sarcopenia-related phenotypes such as lipid accumulation, mitochondrial dysfunction, and oxidative stress, require further investigation.

In conclusion, this study provides multi-level evidence supporting a potential association between PFOS exposure and sarcopenia-related muscle dysfunction. ESRRG and RBP4 were identified as possible mechanistic mediators linking PFOS exposure to impaired myogenic differentiation and altered skeletal muscle homeostasis. By integrating bioinformatics prediction, machine learning, molecular simulation, snRNA-seq analysis, and cellular validation, our findings offer new insights into the potential musculoskeletal toxicity of PFOS and highlight the need for further experimental and epidemiological studies to evaluate its long-term impact on skeletal muscle health.

## Data Availability

The original contributions presented in the study are included in the article/Supplementary Material, further inquiries can be directed to the corresponding author.

## References

[B1] Almohaisen N. Gittins M. Todd C. Sremanakova J. Sowerbutts A. M. Aldossari A. (2022). Prevalence of undernutrition, frailty and Sarcopenia in community-dwelling people aged 50 years and above: systematic review and meta-analysis. Nutrients 14 (8), 1537. 10.3390/nu14081537 35458101 PMC9032775

[B2] Chang C. L. Li Y. R. Wang Z. Y. Li M. L. Jia K. Y. Sun H. X. (2023). Serum retinol binding protein 4 as a potential biomarker for Sarcopenia in older adults. J. Gerontol. A Biol. Sci. Med. Sci. 78 (1), 34–41. 10.1093/gerona/glac151 35857418

[B3] Chen L. K. Arai H. Assantachai P. Akishita M. Chew S. T. H. Dumlao L. C. (2022a). Roles of nutrition in muscle health of community-dwelling older adults: evidence-based expert consensus from Asian Working Group for Sarcopenia. J. Cachexia Sarcopenia Muscle 13 (3), 1653–1672. 10.1002/jcsm.12981 35307982 PMC9178363

[B4] Chen Q. Wang Y. Liu Y. Xi B. (2022b). ATP4B as diagnostic biomarkers for gastric cancer: a bioinformatic analysis based on machine learning. Front. Physiol. 13, 905523, 10.3389/fphys.2022.905523 35812327 PMC9262247

[B5] Chen B. Sun X. Huang H. Feng C. Chen W. Wu D. (2024a). An integrated machine learning framework for developing and validating a diagnostic model of major depressive disorder based on interstitial cystitis-related genes. J. Affect Disord. 359, 22–32. 10.1016/j.jad.2024.05.061 38754597

[B6] Chen Z. Chen Z. Gao S. Shi J. Li X. Sun F. (2024b). PFOS exposure destroys the integrity of the blood-testis barrier (BTB) through PI3K/AKT/mTOR-mediated autophagy. Reprod. Biol. 24 (1), 100846. 10.1016/j.repbio.2023.100846 38160586

[B7] Coperchini F. Awwad O. Rotondi M. Santini F. Imbriani M. Chiovato L. (2017). Thyroid disruption by perfluorooctane sulfonate (PFOS) and perfluorooctanoate (PFOA). J. Endocrinol. Invest 40 (2), 105–121. 10.1007/s40618-016-0572-z 27837466

[B8] Cruz-Jentoft A. J. Sayer A. A. (2019). Sarcopenia. Lancet 393 (10191), 2636–2646. 10.1016/S0140-6736(19)31138-9 31171417

[B9] Fan C. Chen F. Chen Y. Huang L. Wang M. Liu Y. (2024). irGSEA: the integration of single-cell rank-based gene set enrichment analysis. Brief. Bioinform 25 (4), bbae243. 10.1093/bib/bbae243 38801700 PMC11129768

[B10] Freitas A. C. Q. Orsatti C. L. Santato A. S. de Oliveira E. P. Nahas E. A. P. Souza M. V. C. (2024). Retinol-binding protein 4 is a potential biomarker of changes in lean mass in postmenopausal women. Appl. Physiol. Nutr. Metab. 49 (11), 1551–1557. 10.1139/apnm-2024-0156 39377362

[B11] Graham T. E. Yang Q. Blüher M. Hammarstedt A. Ciaraldi T. P. Henry R. R. (2006). Retinol-binding protein 4 and insulin resistance in lean, obese, and diabetic subjects. N. Engl. J. Med. 354 (24), 2552–2563. 10.1056/NEJMoa054862 16775236

[B12] Hao J. Q. Ran B. Hu S. Y. Zhuang Z. X. Zhang J. W. Xiong M. R. (2025). Exploring the link between Di-2-ethylhexyl phthalate (DEHP) exposure and muscle mass: a systematic investigation utilizing NHANES data analysis, network toxicology and molecular docking approaches. Ecotoxicol. Environ. Saf. 295, 118132. 10.1016/j.ecoenv.2025.118132 40194361

[B13] Hardman S. E. Hall D. E. Cabrera A. J. Hancock C. R. Thomson D. M. (2014). The effects of age and muscle contraction on AMPK activity and heterotrimer composition. Exp. Gerontol. 55, 120–128. 10.1016/j.exger.2014.04.007 24747582 PMC4081450

[B14] Hofmann A. Mishra J. S. Yadav P. Dangudubiyyam S. V. Blesson C. S. Kumar S. (2023). PFOS impairs mitochondrial biogenesis and dynamics and reduces oxygen consumption in human trophoblasts. J. Environ. Sci. Public Health 7 (4), 164–175. 37920428 PMC10621633

[B15] Hu X. Wang Z. Zhang L. (2025). Investigating potential targets of Wulingsan in diabetic nephropathy through network pharmacology and experimental validation. Front. Mol. Biosci. 12, 1647796. 10.3389/fmolb.2025.1647796 40800999 PMC12339558

[B16] Huang X. Z. Huang H. Chen H. Wei Y. K. (2024). Identification of endocrine-disrupting chemicals targeting key OP-associated genes via bioinformatics and machine learning. Ecotoxicol. Environ. Saf. 286, 117155. 10.1016/j.ecoenv.2024.117155 39383820

[B17] Jin S. Zheng F. Liu H. Liu L. Yu J. (2025). The correlation between sarcopenia and osteoporosis in the elderly: a systematic review and meta-analysis. Front. Med. (Lausanne) 12, 1603879. 10.3389/fmed.2025.1603879 41103703 PMC12521444

[B18] Kirk B. Zanker J. Duque G. (2020). Osteosarcopenia: epidemiology, diagnosis, and treatment-facts and numbers. J. Cachexia Sarcopenia Muscle 11 (3), 609–618. 10.1002/jcsm.12567 32202056 PMC7296259

[B19] Leek J. T. Johnson W. E. Parker H. S. Jaffe A. E. Storey J. D. (2012). The sva package for removing batch effects and other unwanted variation in high-throughput experiments. Bioinformatics 28 (6), 882–883. 10.1093/bioinformatics/bts034 22257669 PMC3307112

[B20] Liu L. Jiao Y. Yang M. Wu L. Long G. Hu W. (2023). Network pharmacology, molecular docking and molecular dynamics to explore the potential immunomodulatory mechanisms of deer antler. Int. J. Mol. Sci. 24 (12), 10370. 10.3390/ijms241210370 37373516 PMC10299714

[B21] Manickam R. Duszka K. Wahli W. (2020). PPARs and microbiota in skeletal muscle health and wasting. Int. J. Mol. Sci. 21 (21), 8056. 10.3390/ijms21218056 33137899 PMC7662636

[B22] Osorio D. Zhong Y. Li G. Xu Q. Yang Y. Tian Y. (2022). scTenifoldKnk: an efficient virtual knockout tool for gene function predictions via single-cell gene regulatory network perturbation. Patterns (N Y) 3 (3), 100434. 10.1016/j.patter.2022.100434 35510185 PMC9058914

[B23] Perez K. Ciotlos S. McGirr J. Limbad C. Doi R. Nederveen J. P. (2022). Single nuclei profiling identifies cell specific markers of skeletal muscle aging, frailty, and senescence. Aging (Albany NY) 14 (23), 9393–9422. 10.18632/aging.204435 36516485 PMC9792217

[B24] Qin H. Abulaiti A. Maimaiti A. Abulaiti Z. Fan G. Aili Y. (2023). Integrated machine learning survival framework develops a prognostic model based on inter-crosstalk definition of mitochondrial function and cell death patterns in a large multicenter cohort for lower-grade glioma. J. Transl. Med. 21 (1), 588. 10.1186/s12967-023-04468-x 37660060 PMC10474752

[B25] Rangwala S. M. Wang X. Calvo J. A. Lindsley L. Zhang Y. Deyneko G. (2010). Estrogen-related receptor gamma is a key regulator of muscle mitochondrial activity and oxidative capacity. J. Biol. Chem. 285 (29), 22619–22629. 10.1074/jbc.M110.125401 20418374 PMC2903389

[B26] Rao V. K. Das D. Taneja R. (2022). Cancer cachexia: signaling and transcriptional regulation of muscle catabolic genes. Cancers (Basel) 14 (17), 4258. 10.3390/cancers14174258 36077789 PMC9454911

[B27] Sevelsted A. Pedersen C. T. Gürdeniz G. Rasmussen M. A. Schullehner J. Sdougkou K. (2023). Exposures to perfluoroalkyl substances and asthma phenotypes in childhood: an investigation of the COPSAC2010 cohort. EBioMedicine 94, 104699. 10.1016/j.ebiom.2023.104699 37429082 PMC10339117

[B28] Shi M. Wei Y. Zhang W. Wei W. Guo R. Luo F. (2025). Environmental exposome meets muscle wasting: unraveling the VOC-sarcopenia nexus through urinary metabolomics, inflammatory mediation, and network pharmacology in the NHANES cohort. Toxicol. Sci. 206 (2), 373–387. 10.1093/toxsci/kfaf077 40504669

[B29] Shu H. Huang Y. Zhang W. Ling L. Hua Y. Xiong Z. (2023). An integrated study of hormone-related sarcopenia for modeling and comparative transcriptome in rats. Front. Endocrinol. (Lausanne) 14, 1073587. 10.3389/fendo.2023.1073587 36817606 PMC9929355

[B30] Stangl M. K. Böcker W. Chubanov V. Ferrari U. Fischereder M. Gudermann T. (2019). Sarcopenia - Endocrinological and neurological aspects. Exp. Clin. Endocrinol. Diabetes 127 (1), 8–22. 10.1055/a-0672-1007 30199918

[B31] Suman T. Y. Kwak I. S. (2025). Current understanding of human bioaccumulation patterns and health effects of exposure to perfluorooctane sulfonate (PFOS). J. Hazard Mater 487, 137249. 10.1016/j.jhazmat.2025.137249 39842114

[B32] Visser W. J. van de Braak E. E. M. de Mik-van Egmond A. M. E. van der Burgh A. C. de Roos N. M. Jans I. (2023). Effects of correcting metabolic acidosis on muscle mass and functionality in chronic kidney disease: a systematic review and meta-analysis. J. Cachexia Sarcopenia Muscle 14 (6), 2498–2508. 10.1002/jcsm.13330 37728018 PMC10751416

[B33] Wang J. Chen H. Liu Y. Zhou W. Sun R. Xia M. (2015). Retinol binding protein 4 induces mitochondrial dysfunction and vascular oxidative damage. Atherosclerosis 240 (2), 335–344. 10.1016/j.atherosclerosis.2015.03.036 25875385

[B34] Wang W. Gu X. Cao Z. Wang X. Lei Y. Xu X. (2023). A potential correlation between adipokines, skeletal muscle function and bone mineral density in middle-aged and elderly individuals. Lipids Health Dis. 22 (1), 111. 10.1186/s12944-023-01879-z 37525169 PMC10388529

[B35] Wang K. Gan M. Lei Y. Liao T. Li J. Niu L. (2025). Perspectives on mitochondrial dysfunction in the regeneration of aging skeletal muscle. Cell Mol. Biol. Lett. 30 (1), 94. 10.1186/s11658-025-00771-1 40721743 PMC12305941

[B36] Widner D. B. Liu C. Zhao Q. Sharp S. Eber M. R. Park S. H. (2021). Activated mast cells in skeletal muscle can be a potential mediator for cancer-associated cachexia. J. Cachexia Sarcopenia Muscle 12 (4), 1079–1097. 10.1002/jcsm.12714 34008339 PMC8350201

[B37] Yan X. Zhou J. Cao X. (2025). Risk factors of frailty/sarcopenia in community older adults: Meta-analysis. Open Med. (Wars) 20 (1), 20251259. 10.1515/med-2025-1259 40989160 PMC12452068

[B38] Yuan S. Larsson S. C. (2023). Epidemiology of sarcopenia: prevalence, risk factors, and consequences. Metabolism 144, 155533. 10.1016/j.metabol.2023.155533 36907247

[B39] Zhang Y. Hao Q. Ge M. Dong B. (2018). Association of sarcopenia and fractures in community-dwelling older adults: a systematic review and meta-analysis of cohort studies. Osteoporos. Int. 29 (6), 1253–1262. 10.1007/s00198-018-4429-5 29500527

[B40] Zhang K. Z. Li J. W. Xu J. S. Shen Z. K. Lin Y. S. Zhao C. (2024a). RBP4 promotes denervation-induced muscle atrophy through STRA6-dependent pathway. J. Cachexia Sarcopenia Muscle 15 (4), 1601–1615. 10.1002/jcsm.13518 39031684 PMC11294031

[B41] Zhang B. Yang Y. Li Q. Ding X. Tian M. Ma Q. (2024b). Impacts of PFOS, PFOA and their alternatives on the gut, intestinal barriers and gut-organ axis. Chemosphere 361, 142461. 10.1016/j.chemosphere.2024.142461 38810808

[B42] Zuo X. Tan S. Zhang Y. Zhang C. Ma L. Hou X. (2025). Linking PFOS exposure to chronic kidney disease: a multimodal study integrating epidemiology, network toxicology, and experimental validation. Ecotoxicol. Environ. Saf. 302, 118770. 10.1016/j.ecoenv.2025.118770 40737851

