# Supplementary material for: Multi-omics integration reveals the role of PFOS in sarcopenia via ESRRG and RBP4: from computational prediction to experimental validation
Source: Front Pharmacol. 2026 Oct 8;17:1926144. doi: 10.3389/fphar.2026.1926144 (PMC13686843; doi:10.3389/fphar.2026.1926144)
Supplement: Supplementary file 1 [file Table1.docx]

Table S1 Relevant information concerning the dataset

| GSE series | Sample | Source types | Data type | Platform | Group |
| --- | --- | --- | --- | --- | --- |
| GSE25941 | 15 young people and 21 older people | human muscle | Bulk transcriptome | GPL570 | Discovery cohort |
| GSE38718 | 14 young people and 8 older people | human muscle | Bulk transcriptome | GPL570 | Discovery cohort |
| GSE9103 | 10 young people and 10 older people | human muscle | Bulk transcriptome | GPL570 | Discovery cohort |
| GSE1428 | 10 young people and 12 older people | human muscle | Bulk transcriptome | GPL96 | Validation cohort |
| GSE167186 | 6 young and 11 older people | human skeletal muscle | Single-nucleus RNA-seq | GPL24676 | snRNA-seq |
